# Effect of Carnitine and herbal mixture extract on obesity induced by high fat diet in rats

**DOI:** 10.1186/1758-5996-1-17

**Published:** 2009-10-16

**Authors:** Kamal A Amin, Mohamed A Nagy

**Affiliations:** 1 Biochemistry Department Faculty of Veterinary Medicine, Beni-Suef University, Beni-Suef, Egypt; 2 Chemistry Department Faculty of Science Beni-Suef, Beni-Suef University, Beni-Suef, Egypt

## Abstract

**Background:**

Obesity-associated type 2 diabetes is rapidly increasing throughout the world. It is generally recognized that natural products with a long history of safety can modulate obesity.

**Aim:**

To investigate the development of obesity in response to a high fat diet (HFD) and to estimate the effect of L-carnitine and an Egyptian Herbal mixture formulation (HMF) (consisting of T. chebula, Senae, rhubarb, black cumin, aniseed, fennel and licorice) on bodyweight, food intake, lipid profiles, renal, hepatic, cardiac function markers, lipid Peroxidation, and the glucose and insulin levels in blood and liver tissue in rats.

**Method:**

White male albino rats weighing 80-90 gm, 60 days old. 10 rats were fed a normal basal diet (Cr), 30 rats fed a high-fat diet (HFD) for 14 weeks during the entire study. Rats of the HFD group were equally divided into 3 subgroups each one include 10 rats. The first group received HFD with no supplement (HFD), the 2^nd ^group HFD+L-carnitine and the third group received HFD+HMF. Carnitine and HMF were administered at 10^th ^week (start time for treatments) for 4 weeks.

Body weight, lipid profile & renal function (urea, uric acid creatinine) ALT & AST activities, cardiac markers, (LDH, C.K-NAC and MB) the oxidative stress marker reduced glutathione (GSH), and Malondialdehyde (MDA) catalase activity, in addition to glucose, insulin, and insulin resistance in serum & tissues were analyzed.

**Results:**

Data showed that feeding HFD diet significantly increased final body weight, triglycerides (TG), total cholesterol, & LDL concentration compared with controls, while significantly decreasing HDL; meanwhile treatment with L-carnitine, or HMF significantly normalized the lipid profile.

Serum ALT, urea, uric acid, creatinine, LDH, CK-NAC, CK-MB were significantly higher in the high fat group compared with normal controls; and administration of L-carnitine or herbal extract significantly lessened the effect of the HFD. Hyperglycemia, hyperinsulinemia, and high insulin resistance (IR) significantly increased in HFD in comparison with the control group. The treatment with L-carnitine or HMF improved the condition. HFD elevated hepatic MDA and lipid peroxidation associated with reduction in hepatic GSH and catalase activity; whereas administration of L-carnitine or herbal extract significantly ameliorated these hepatic alterations.

**Conclusion:**

HFD induced obesity associated with a disturbed lipid profile, defective antioxidant stability, and high values of IR parameters; this may have implications for the progress of obesity related problems. Treatment with L-carnitine, or HMF extract improved obesity and its associated metabolic problems in different degrees. Also HMF has antioxidant, hypolipidaemic insulin sensitizing effects. Moreover HMF might be a safe combination on the organs whose functions were examined, as a way to surmount the obesity state; and it has a distinct anti-obesity effect.

## Introduction

The global prevalence of obesity is increasing rapidly among adults as well as among children and adolescents in places where high dietary fat intake is a major risk factor for the development of obesity [1]. Obesity is reaching epidemic proportions worldwide; it is correlated with various comorbidities, among which the most relevant are dyslipidemia [2], diabetes mellitus T2DM [3], fatty liver (which can later progress to nonalcoholic fatty liver disease [4]), cardiovascular (CV) diseases such as heart failure (HF) and coronary heart disease (CHD) [5].

L-carnitine is essential for the transfer of long-chain fatty acids from the cytosol to mitochondria for subsequent beta oxidation. Its lack impairs the ability to use fat as fuel. This can result in an acute metabolic decompensation, most often early in life, with hepatic encephalopathy and hypoketotic hypoglycemia [6].

Carnitine (L-beta-hydroxy-gamma-N,N,Ntrimethyl-aminobutyric acid) is one of the "nutraceuticals" that has pleiotropic biologic effects. L-carnitine administration to carnitine-deficient rats led to normalization in myocardial function including indices of contractility relaxation, systolic diastolic blood pressure [7].

The liver is a central organ for carnitine metabolism; therefore it is not surprising that carnitine metabolism is impaired in patients and experimental animals with certain types of chronic liver disease. L-carnitine can have a therapeutic role in some of these conditions [8].

Nowadays there is an increased demand for using plants in therapy "back to nature" instead of using synthetic drugs which may have adverse effects. Traditional medicinal plants are often cheaper, locally available, and easily consumable (raw or as simple medicinal preparations). These simple medicinal preparations often mediate beneficial responses due to their active chemical constituents.

The reason for using herbs in combination is that herbs have chemicals components which can bring strong effects on body. When herbs are used in combination it helps the body better manage potentially undesirable effects of any one and each herb in the combination/formulation plays a curative or pacifying role. It is therefore preferable to use herbal combinations instead of depending on single herbs [9].

The HMF contains antioxidants, stimulant laxatives that are present in T. chebula, senna, licorice and rhubarb. The herbal supplement also includes carminatives which are represented by black cumin, aniseed, fennel, and licorice [10]. These carminative herbs are utilized to improve digestion or to treat dyspepsia or irritable bowel symptoms of ulcerative colitis; and to treat maladies of specific organs in the digestive system, such as the pancreas, liver, stomach or large and small intestines.

Demulcents and carminatives are often used to soften and physiologically balance the harsh effects of stimulant laxatives [11]. Both licorice and aniseed are considered to be flavors that by altering the characteristics of the solute, causing sweet and sour tanginess.

The ethanolic extract of roots of rhubarb led to the isolation of anthraquinones: chrysophanol, physcion, emodin, glucopyranoside, stilbenes: desoxyrhaponticin, rhaponticin, resveratrol, rhapontigenin, glucopyranoside, ampelopsin B and rhaponticin [12].

The mechanism of rhubarb action is twofold: 1) stimulation of colonic motility, which augments propulsion and accelerates colonic transit which in turn reduces fluid absorption from the fecal mass; 2) an increase in the paracellular permeability across the colonic mucosa, probably owing to an inhibition of Na^+^/K^+ ^exchanging ATPase or to an inhibition of chloride channels. This results in an increase in the water content in the large intestine. Purgation is followed by an astringent effect owing to the tannins presence [13].

Licorice root is the dried peeled or unpeeled root of *Glycyrrhiza glabra *known as Spanish licorice; or of other varieties of *Glycyrrhiza glabra *[14].

Constituents with antioxidant capacity were isolated from *Glycyrrhiza glabra*. The isolated compounds were identified as the isoflavans, Hispaglabridin A, Hispaglabridin B, Glabridin; the two chalcones, isoprenyl chalcone derivative isoliquiritigenin; theisoflavone, formononetin. Among these compounds, Glabridin constituted the major component in the crude extract and the most potent antioxidant toward LDL oxidation [15].

Glycyrrhizin inhibited histamine release from rat mast cells prevented carbon Tetrachloride-induced liver lesions and macrophage-mediated cytotoxicity. Glycyrrhizin protected the liver apparently through its membrane stabilization effects [16]. Glycyrrhizic acid its glucoside, glycyrrhizin impart the unique licorice taste. Glycyrrhizin is 50 times sweeter than sucrose. Licorice sweetness has a slower onset than sugar so it is important for the palatability of the HMF.

Aniseed is the dried ripe fruits of the *Pimpenella anisum *family, containing licorice-like components, *anise *which is sweet, very aromatic and anethole, are the principal component of anise oil [17].

Fennel is the ripe fruit, or seed, of *Foeniculum vulgare*. It has a strongly aromatic odor somewhat bitter sweet pungent taste. The British Herbal Pharmacopoeia reported its action as a carminative is considered to be one of the best additions to purgative medicines, and it is often compounded with T. chebula, senna and rhubarb, in infusions or mixture [18]. Fennel seed extracts may possess a radical scavenging antioxidant activity due to the occurrence of some phenol compounds in fennel being responsible for such an activity [19].

Black cumin is the dried ripe seeds of Nigella sativa (NS), strongly aromatic when crushed, reminiscent of anise or nutmeg, also slightly bitter tasting at first, then spicy and somewhat pungent. It contains fixed, volatile oils which contain thymoquinone and several monoterpenes, including p-cymene a-pinene [20]. Many therapeutic effects of NS extracts have been documented, including immunomodulative, anti-inflammatory, antitumor, antidiabetic and antiulcerogenic [21] effects in both clinical and experimental studies.

Recently, the use of powerful drugs has become a popular means to overcome excess weight. Adverse effects may limit their overall usefulness. Accordingly, recent preliminary reports suggesting that herbs with along history of use of other natural substances is less likely to produce toxicity and might be effective in reducing food intake, promoting significant weight loss, are encouraging. Preliminary findings suggest that, at least, one formulation of herbs has such promise.

However, the effect of HMF on lipid peroxidation and antioxidant enzymes activities in obesity has not been examined and so far, little is known on the medicinal values of HMF. Thus, the present investigation was carried out in order to study the possible antiobesity, hypolipidemic, hypoglycemic and antioxidant effect of HMF. The effect was compared with L-carnitine in a model of high fat diet-induced obesity. Moreover, the extract was also tested for its hepatic cardiac and renoprotective effects in rats.

## Material and Methods

This study was approved by the Committee of Scientific Ethics at Beni Suef University

### Materials

#### 1- Diets

composition of the experimental diet (g/kg diet) was according to the formula of **Kim et al**. [22]. It included the normal diet for control rats (fat 5%, carbohydrates 65%, proteins, 20.3% fiber 5%, salt mixture and 3.7% vitamin mixture 1%). The high fat diet contained fat 46%, carbohydrates 24%, proteins, 20.3%, fiber 5%, salt mixture 3.7%, vitamin mixture 1%. Normal and HFD constituents were purchased from El-Gomhoria Company, Cairo, Egypt. HFD was preserved at 4°C until used.

#### 2- Experimental animals

40 white male albino rats weighing 80-90 gm, 60 day old were used for this study. They were purchased from the National Research Center, Cairo, Egypt. All animals were housed in stainless steel cages contain barriers for each rat for individual housing and the cage contain 6 rats and each rat had a tag number. They kept under standard environmentally controlled, clean-air room with temperature 24 ± 5°C, illumination (12 h light/12 h dark cycles), a relative humidity of 60 ± 4%, and water and rodent chow were available ad libitum throughout the period of the investigation. They were housed for two weeks after their arrival in the laboratory for accommodation.

Our work was carried out in accordance with the guidelines of Faculty of Science at Beni Suef University for animal use. These animals were used for induction of obesity.

Food consumption was calculated daily at the same time by subtracting the amount of food left over in each cage barrier for each rat from the measured amount of food provided at the previous day (gm/day/rat). The mean of food consumption per each rat was considered by dividing the amount of food eaten in a week by 7.

The average of food consumptions were represented in gm/day/rat and the body weight for each rat was determined once a week (g).

#### 3- Drug administration

**L-carnitine **(dietary supplement): 1 ml containing 250 mg carnitine was purchased from the Arab Company for Pharmaceuticals Medicinal Plants (MEPACO, Egypt (Enshas El Raml-Sharkeiya). Oral administration of a dose of 250 mg/kg daily was done according to the method described by **Oka et al **[23]. Handling of the animal was the same for all groups and did not affect weight gain.

#### 4- Plant material preparation of the herbal formulation

Herbs were purchased from local Mohey El-Attar Company in *El-Minia *city. Identification and extractions of medicinal plants were completed in department of Pharmacognacy, faculty of pharmacy, *El Minia *University.

The preparation, composition dose of the herbal formula of each herbal extract in the HMF and the identification of their main chemical groups were realized as previously described as follow: Rhubarb 750 mg/kg body weight according to **Kang Jin **[24], senna (*Cassia angustifolia*) 750 mg/kg body weight [25], T. chebula 750 mg/kg body weight [26], Sweet fennel 300 mg/kg body weight [11], Aniseed 10 mg/kg body weight [27], licorice (*Glycyrrhiza galabra*) 300 mg/kg body weight [15] Black cumin (Nigella sativa) 300 mg/kg body weight [28].

The extraction was done by water extract for T. chebula, ethanol for senna and Nigella sativa, ethanol-water extract for rhubarb, licorice, *Pimpenella anisum *and *Foeniculum vulgare *(fennel). The extract of each plant was collected and the mixture administered orally as a suspension by stomach tube at a dose of 790 mg/kg body weight daily, the volume of the vehicle being kept constant at 1 ml/kg.

The identified compounds belonged to some specific structural types: flavenoids glabridin, and sennosides. After isolation by several column chromatographic steps from the extract and characterization by spectroscopic methods, the main compounds were identified as triterpenoids, coumarin, gallic acid, chebulin and ellagic acid as well as other phenolic compounds from T. chebula extraction.

Anthraquinones, rhein, and tannins were isolated from rhubarb extraction. Glabridin from *Glycyrrhiza galabra *extraction, thymoquinone from Nigella sativa extraction. Phenolic content, d-limonene β-myrcene from *Foeniculum vulgare *(fennel) extraction and anethole form Pimpenella anisum extract.

### Methods

#### 1-Experimental design and animal grouping

A total 40 rats were randomly assigned into two groups, normal 10 rats and obese 30 rats. Obesity was induced in rats for 70 day by feeding the high fat diet. The rats were included in four groups after induction of obesity. In the experiment 10 rats were used in each group.

• **Normal group**, 10 rats fed on normal diet during the entire study (98 day).

• **Obese group**, 10 rats given high-fat diet during the entire study (98 day) and saline oral daily by using stomach tube at 10^th ^week.

• **HFD+carnitine group**, 10 obese rats received HFD during the entire study and 250 mg/kg L-carnitine for 28 days as a single daily dose in the morning.

• **HFD+ HMF group**, 10 obese rats received HFD during the entire study and 790 mg/kg mixed herbal extract for 28 days as single oral daily by using stomach tube.

Our goal is to achieve obesity model in 10 weeks following by treatment period for 4 weeks. This model provided us reliable method and resembles the clinical cases of obesity and its treatments; also this period of treatment is safe and recommended in previous research.

#### 2- Sampling and tissue preparation

##### Blood Sampling

By the end of the experimental periods, venous blood samples were collected from the orbital sinus of normal, obese control, obese treated rats via glass capillaries at fasting state. The blood samples were collected in dry glass centrifuge tubes, allowed to coagulate at room temperature and centrifuged at 3500 rpm for 15 minutes at room temperature for separation of serum.

The clear, non-haemolysed supernatant sera were separated using clean dry disposable plastic syringes and stored at -20°C for subsequent biochemical measurements as follows: lipid profile, liver enzyme activities related to its function, kidney function, heart biomarkers, glucose, insulin concentration, and oxidative stress markers.

##### Tissue samples

Rats were sacrificed by decapitation and an abdominal incision was immediately done for separation of hepatic, perirenal, and visceral adipose tissues. These were dried on filter paper and weighed (g.). The liver was immediately excised and weighed (g.) and underwent homogenization for GSH & MDA catalase measurements.

#### 3- Biochemical analysis of Serum and tissue

Serum glucose was estimated according to the method of **Trinder **[29] using Stanbio Laboratory USA Kits. Serum insulin was assayed in the Radioactive Isotopes Unit, Central Department of Scientific Analysis and Testing, National Research Center (Dokki, Giza) using radioimmunoassay kits (Diagnostic Products Corporation, Los Angeles, USA) [Coat-A-Count] according to the method of **Marschner et al**. [30]. Insulin resistance was calculated using the Homeostasis Model Assessment [31]. ALT and AST activities were measured according to the method of **Reitman et al**., [32] using kits purchased from Rox Company, United Kingdom.

Serum urea and creatinine levels were measured colorimetrically [33]**; **serum uric acid was measured according to the method of **Sanders et al.**[34] using kits purchased from Diamond Diagnostic Egypt. Serum was analyzed for total cholesterol [35], triglycerides [36], VLDL [37], and HDL [38], by enzymatic colorimetric methods using kits.

Serum was evaluated for CK-MB [39] CK-NAC and LDH activity [40]. Liver lipid peroxidation was measured through Malondealdehyde (MDA) levels, according to the methods of **Mihara and Uchiyama **[41]. Liver glutathione content and catalase (CAT) activity was determined according to the procedure of **Beutler et al**. [42] and **Cohen et al**. [43] respectively.

### Statistical analysis

Statistical analysis was carried out using Graph Pad Instat software (version 3, ISS-Rome, Italy). Unless otherwise specified, groups of data were compared with an unpaired t-test one-way analysis of variance (ANOVA) followed by Tukey-Kramer (TK) multiple comparisons post-test. Values of P < 0.05 were regarded as significant. Data were expressed in tables and figures as mean ± standard error (SEM).

## Results

**Figure 1:**
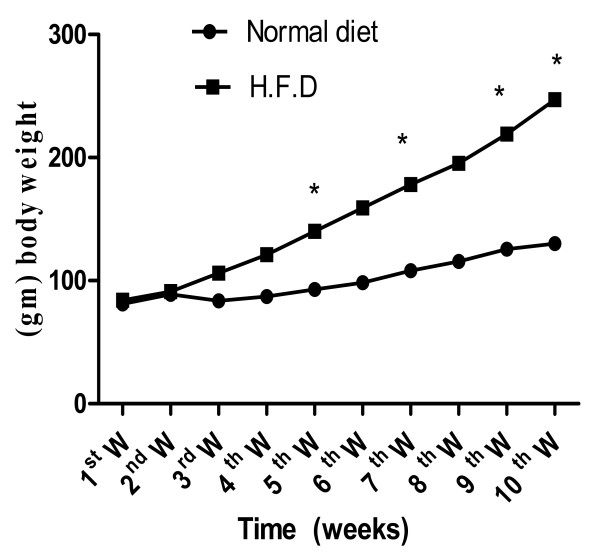
**Effect of normal and HFD on whole body weights weekly during 10 weeks in rats**. Body weight increased significantly in rats fed on the HFD during 5^th^, 7^th^, 9^th ^and 10^th ^W. compared with controls. Values significantly different compared to normal *P < 0.05.

**Figure 2:**
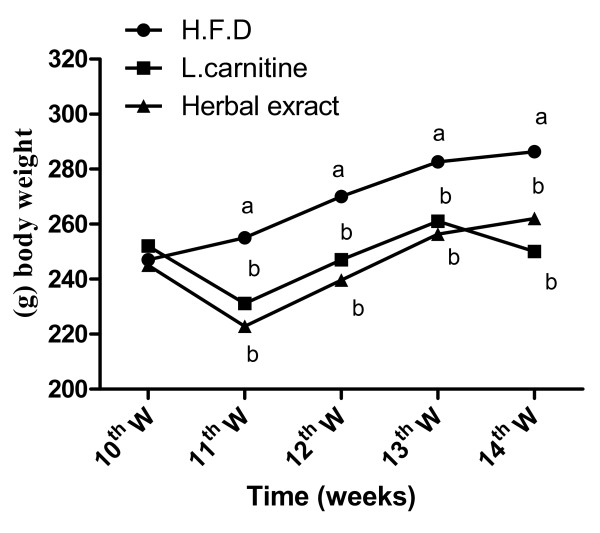
**Effect of HFD, L-carnitine and HMF on body weights during treatment period in rats**. Treatments with L-carnitine or HMF significantly reduced the elevated body weight during the treatment period compared to HFD. (HFD vs. L carnitine or HMF) Means not sharing common letter are significantly different (p < 0.05).

Body weight increased significantly in rats on the HFD compared with controls (Figure 1), while treatments with L-carnitine or HMF significantly reduced this gain during the treatment period (Table 1 and figure 2).

**Table 1:** Effect of HFD, L-Carnitine and HMF on whole body weights of rats.

**Weeks**	**HFD**	**L. carnitine**	**HMF**
**11^th ^Week**	255.0 ± 3.54^a^	231.2 ± 2.56^b^	222.8 ± 3.58^b^

**12^th ^Week**	270.0 ± 2.60^a^	247.0 ± 3.36^b^	239.6 ± 4.19^b^

**13^th ^Week**	282.6 ± 2.09^a^	261.0 ± 3.81^b^	256.4 ± 5.07^b^

**14^th ^Week**	286.4 ± 3.97^a^	250.0 ± 3.41^b^	262.0 ± 6.04^b^

Values significantly different compared to normal P** < 0.01. Values are expressed as means ± SEM. Means not sharing common letter are significantly different (p < 0.05).

**Figure 3:**
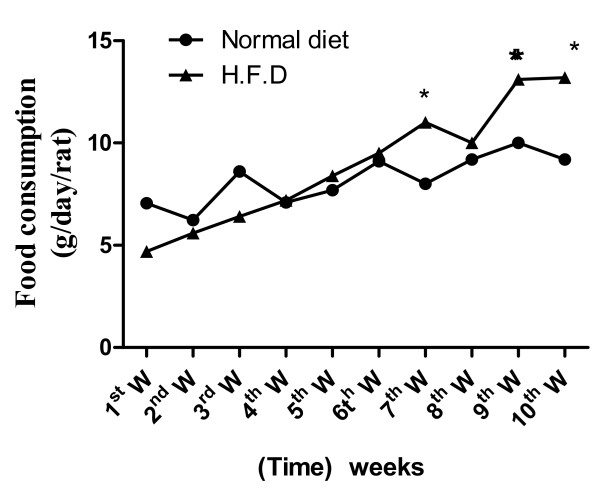
**Effect of normal and HFD on food consumption during 10 weeks in gm/day/rat**. Food consumption increased significantly in the HFD group during 7^th^, 9^th ^and 10^th ^W. compared with controls. Values significantly different compared to normal *P < 0.05.

**Figure 4:**
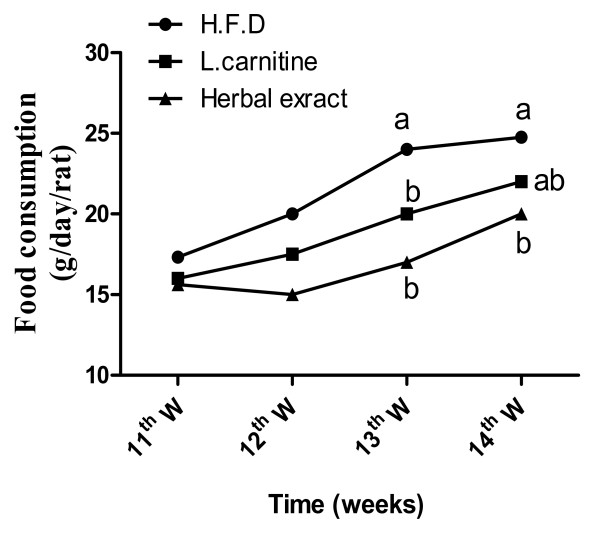
**Effect of HFD, L-carnitine and HMF on food consumption during treatment period gm/day/rat**. Treatments with L-carnitine or HMF significantly ameliorated the changes during 13^th ^and 14^th ^W. of the treatment period (HFD vs. L carnitine or HMF). Means having different letter are significantly different (p < 0.05).

Food consumption increased significantly in the HFD group compared with controls (Figure 3), while treatments with L-carnitine or HMF significantly ameliorated the changes during the treatment period (Table 2 and figure 4).

**Table 2:** Effect of HFD, L-Carnitine and HMF on food intake in rats

**Weeks**	**HFD**	**L. carnitine**	**HMF**
**11^th ^Week**	17.33 ± 1.05^a^	16 ± 1.58^a^	15.63 ± 0.62^a^

**12 **^st^**Week**	20 ± 1.58^a^	17.50 ± 1.32^a^	15 ± 0.71^a^

**13^th ^Week**	24 ± 2.27^a^	20 ± 1.63^b^	17 ± 1.23^b^

**14^th ^Week**	24.75 ± 2.29^a^	22 ± 1.83^a, b^	20 ± 1.47^b^

Values significantly different compared to normal P** < 0.01. Values are expressed as means ± SEM. Means not sharing common letter are significantly different (p < 0.05).

Serum TG, cholesterol, LDL/VLDL levels, and perirenal and mesenteric fat were significantly elevated in the HFD group, while HDL was significantly decreased compared to the controls. Additional administration of L-carnitine or HMF significantly improved these changes (Table 3).

**Table 3:** Effect of HFD, L-Carnitine and HMF on plasma lipid profile and adipose tissues weight in HFD rats

	**Normal diet**	**HFD**	**L-Carnitine**	**HMF**
**TG (mg/dl)**	63.94 ± 2.19	172.6 ± 1.73**^a^	118.5 ± 1.21 b	116 ± 1.83^b^

**VLDL (mg/dl)**	12.79 ± 0.44	34.52 ± 0.35**^a^	23.69 ± 0.24^b^	23.20 ± 0.37^b^

**T.C (mg/dl)**	95.29 ± 1.03	152.7 ± 2.56**^a^	109.6 ± 1.51^b^	113.1 ± 1.54^b^

**HDL (mg/dl)**	70.97 ± 1.89	53.34 ± 2.52**^a^	67.45 ± 1.4^b^	65.59 ± 1.36^b^

**LDL (mg/dl)**	12.48 ± 2.0	67.06 ± 1.65**^a^	17.29 ± 0. 85^b^	17.42 ± 2.08^b^

**Visceral fat (gm)**	3.54 ± 0.51	14.8 ± 1.07**^a^	10.10 ± 0.33^b^	10.40 ± 0.93^b^

**Perirenal fat (gm)**	3.54 ± 0.51	14.8 ± 1.07**^a^	10.10 ± 0.33 b	10.40 ± 0.93^b^

Values significantly different compared to normal P** < 0.01. Values are expressed as means ± SEM. Means not sharing common letter are significantly different (p < 0.05)

Serum LDH, CK, AST, ALT, urea, uric acid, and creatinine concentrations were significantly higher in the HFD group compared to the control one; these changes, were affected by supplementation with L-carnitine and HMF (Table 4).

**Table 4:** Effect of HFD, L-Carnitine and HMF on cardiac biomarkers, liver enzyme activity and kidney function tests of HFD rats

	**Normal diet**	**HFD**	**L-Carnitine**	**HMF**
**LDH** **(u/I)**	258.5 ± 10.8	541.7 ± 10.62**^a^	472.8 ± 6.4^b, c^	497.9 ± 8.23^b^

**C.K-NAC** **(u/I)**	250.2 ± 12.6	457.7 ± 10.58**^a^	407.4 ± 8.9^b^	396.4 ± 8.92^b, c^

**C.K-MB (u/I)**	130.0 ± 5.03	262.0 ± 9.55**^a^	213.8 ± 3054^b^	209.8 ± 8.95^b^

**AST (u/I)**	24.94 ± 0.95	56.8 ± 1.09**^a^	41.18 ± 1.11^b^	44.24 ± 1.09^b^

**ALT (u/I)**	17.14 ± 0. 93	5.37 ± 0.95**^a^	24.60 ± 0.69^b^	25.67 ± 0.95^b^

**Urea (mg/dl)**	36.60 ± 0.94	50.11 ± 1.33**^a^	40.86 ± 0.71^b, d^	42.36 ± 0.72^b^

**Uric acid (mg/dl)**	2.33 ± 0.07	5.92 ± 0.08**^a^	4.94 ± 0.07^b^	5.13 ± 0.05^b^

**Creatinine (mg/dl)**	0.59 ± 0.05	1.31 ± 0.06**^a^	0.96 ± 0.02^b^	1.01 ± 0.03^c^

Values significantly different compared to normal P** < 0.01. Values are expressed as means ± SEM. Means not sharing common letter are significantly different (p < 0.05).

Serum levels of glucose, insulin and hepatic MDA, were significantly raised, while hepatic GSH and catalase were significantly lowered in the HFD group compared to the controls. These alterations were ameliorated by administration of L-carnitine HMF but there were no differences between L-carnitine and HMF (Table 5).

**Table 5:** Effect of diet and treatments on serum glucose, insulin, insulin resistance lipid peroxidation in obese rats

	**Normal diet**	**HFD**	**L-Carnitine**	**HMF**
**Glucose (mg/dl)**	57.87 ± 1.96	145.1 ± 2.26**^a^	120.9 ± 1.96^b, c^	118.0 ± 3.23^c, d^

**Insulin** **(μIU/ml)**	4.40 ± 0.15	6.69 ± 0.13**^a^	4.5 ± 0.07^b^	4.41 ± 0.04^b^

**Insulin resistance**	1.17 ± 0.09	2.89 ± 0.07**^a^	2.46 ± 0.09^b^	2.40 ± 0.05^b^

**M.D.A (n mol M.D.A/g/hr)**	4.69 ± 0.06	9.72 ± 0.08**^a^	7.96 ± 0.08^b, c^	8.21 ± 0.07^b^

**GSH (n mol/** **100 mg tissue)**	72.65 ± 0.72	57.31 ± 0.72**^a^	68.77 ± 0.89^b, c^	68.45 ± 0.46^b^

**Catalase (K×10-2)**	52.60 ± 0.96	19.88 ± 0.63**^a^	36.97 ± 0.71^b^	32.22 ± 1.06^c^

Values significantly different compared to normal P** < 0.01. Values are expressed as means ± SEM. Means not sharing common letter are significantly different (p < 0.05).

## Discussion

### Effect of diet treatments on body weight food consumption

Obesity is considered to be a disorder of energy balance, occurring when energy expenditure is no longer in equilibrium with daily energy intake, so as to ensure body weight homeostasis [44]. Although the etiology of obesity is complex, dietary factors, particularly the consumption of a HFD, is considered a risk factor for its development [45].

The current results showed that body weight increased significantly in the HFD group compared with the normal group (Figure 1), a result in accordance with that of **Xu et al **[46]; this is associated with increased food intake.

Consumption of the HFD led to obesity because it facilitates the development of a positive energy balance leading to an increase in visceral fat deposition; this led to abdominal obesity in particular. Moreover, **Schrauwen-Hinderling et al **[47] found that HFD feeding is accompanied by molecular adaptations that favor fat storage in muscle rather than oxidation.

In the current study, rats fed HFD consumed considerably more food than the control rats throughout the experiment (Table 2 and Figure 3). So their caloric intake was increased and they showed a large increase in perirenal visceral adipose tissue mass (Table 3), suggesting that the excess energy led to the build up of adiposity. This is the source of the increase in body weight. Rat consuming the high fat ration actually received about 27% more kilocalories, more weight, and had larger fat pads than rats fed only chow. Rats consuming a palatable dietary fat in addition to a standard chow diet take in about 10% more calories a day than rats fed only the chow diet and over time, become obese and exhibit a number of complications of obesity.

Our results showed a significant decrease in food intake, whole body weight, and adipose tissue accumulation from oral administration of L-carnitine (Tables 1, 2). Interest in the role of L-carnitine as a feed additive to improve whole body composition arose from the desire to partition nutrients away from lipid accretion, causing improvement of nitrogen balance; L-carnitine also attenuated visceral fat accumulation and accelerated the normalization of food intake [48].

The current results due to the effect of HMF, showed a significant decrease in body weight and food consumption (Table 1, 2) in accordance with the findings of **York et al **[49]. Several isoflavans constituents of licorice are unique phytoestrogens, which like estradiol, affect the serotonergic system, inhibiting serotonin re-uptake and thereby increasing the levels of serotonin in synaptic clefts. This enhances satiety and resembles the action of sibutramine, but naturally [50].

Dietary phytoestrogens may also activate AMPK leading to a reduction in food intake [51]. Our study is a novel trial for using a phytoestrogen in fennel, licorice and anise as a natural serotonin reuptake inhibitor instead of sibutramine.

Anetholein fennel as a carminative herb improves the digestion by stabilization of the gastrointestinal mucous membrane and causing the pancreas to increase its secretions [11]. Hydroxyl-anthracene glucosides, especially sennosides A B in senna and T. chebula of HMF improve gastrointestinal motility and influence colonic motility thereby reducing fluid absorption and facilitate weight loss.

### Effect of diet treatments on serum lipid profile in rats

In the present study, a high fat diet resulted in dyslipidemic changes as illustrated by increasing triglycerides, VLDL, total cholesterol and low density lipoprotein LDL and a decrease in serum level of high density lipoprotein HDL (Table 3) a finding in accordance with that of **Woo et al**. [52].

L-carnitine supplementation produced significant decreases in serum TG, VLDL, and T-cholesterol, LDL-C while there were significant increases in HDL cholesterol in obese rats. These results are in agreement with those of **González-Ortiz et al. **[53] and **El-Metwally et al**., [54] who reported that oral L-carnitine increases plasma free carnitine levels, improves dyslipidemia and decreases oxidative stress, with reduction of cardiac parameters.

L-carnitine administration to obese rats reduces significantly serum hypertriglyceridemia (Table 3) via decreased synthesis of triglycerides by the liver or by inhibition of triglyceride release from the liver. Also L-carnitine induced marked reductions in total serum cholesterol in skeletal muscles of obese rats [55].

L-carnitine is necessary for mitochondrial transport metabolism of long-chain fatty acids, thus for myocardial energetic metabolism. Fatty acids cross mitochondrial membranes as acyl-carnitine derivatives to enter pathways for oxidation, acylation, chain shortening or chain elongation-desaturation. Therefore, L-carnitine-dependent fatty acid transfer is central to lipid metabolism; dietary supplementation of L-carnitine improves the utilization of fat providing marked reduction in plasma levels of TG [56].

In our experimental rat model of obesity, treatment with HMF led to significant decrease in TG, VLDL, T-cholesterol and LDL-C but a significant increase in HDL cholesterol (Table 3), a result in agreement with that of **Murali et al **[57].

T. chebula components of HMF have hypocholesterolemic activity that might be mediated through increased cholesterol excretion in the feces. In addition anthraquinone glycosides from rhubarb in the HMF have lipid-lowering effects, resulting in depression of lipid accumulation. It consequently has anti-atherosclerotic properties [58].

Glycyrrhizin, the active constituent in licorice root, which is another plant in the mixture, exerts hypocholesterolemic action by stimulating the conversion of cholesterol into bile acids without effect on cholesterol synthesis [59]. Another herbal supplement in the HMF, Nigella sativa, contains active constituents, phytosterols (mostly b-sitosterol, stigmasterol campesterol) with the ability to reduce the intestinal absorption of diet biliary cholesterol [60].

### Effect of diet treatments on cardiac biomarkers in rats

The obese rats showed a significant increase in the activity of serum LDH, CK-NAC and CK-MB when compared to the normal rats (Table 4). Similar results were reported by **Diniz et al. **[61]. Reduced muscle mitochondrial content function with increasing obesity would lower the total cellular ATP yield, which would result most notably in increased mitochondrial volume, and increased glycolytic enzymes necessitating increased activity of creatine kinase, as this enzyme is responsible for rapidly transferring high-energy phosphate groups from the site of production to the site of use [62].

The increased blood levels of total cholesterol, LDL, VLDL as well as lowered levels of HDL in HFD rat have been identified in the development of hypercholestremia, which is one of the risk factors for CAD [63]. Administration of L-carnitine produces a significant decrease in the activity of CK-NAC, CK-MB and LDH (Table 4). This is in agreement with **Ferrari et al**. [64], who reported a reduction in cardiac markers, with L-carnitine having a good protective effect on myocardium.

In conclusion, L-carnitine stimulates the activity of pyruvate dehydrogenase (PDH) which is an important enzyme catalyzing the rate-limiting step in lactate utilization. In type 2 diabetes this led to significant decrease in the activity of LDH [65]. Moreover, L-carnitine in the β-oxidation of fatty acid as parallel source for energy acts synergistically with the creatine/phosphocreatine/creatine kinase system to produce significantly decreased activity of creatine kinase.

This effect is mainly attributable to the vasodilatation property of L-carnitine, which both improves energetic metabolism of the hypoxic/damaged muscle and enhances wash-out of algogenic metabolites.

Our findings showed that obese rats treated with the HMF exhibited significant decreases in LDH, CK-NAC and CK-MB activity (Table 4), in with the findings of **Suchalatha et al**. [66], who reported that T. chebula extract treatment ameliorates the effect of lipid peroxide formation that is related to the activities of diagnostic myocardial marker enzymes.

The rhubarb content in our mixture could prevent the development of atherosclerosis through regulating vascular inflammatory processes in rats fed with an atherogenic diet [67]; and the thymoquinone content in *N. sativa *normalized cardiac histopathology, as it decreased lipid peroxidation [20,21].

### Effect of treatments on renal function tests of HFD in rat

The obese rats showed a highly significant increase in the concentration of serum urea, uric acid creatinine, compared with the normal group (Table 4) which is in agreement with the results of **Cindik et al. **[68].

Abnormal renal function is mainly associated with diabetic nephropathy. The pathophysiology involves glucose that binds irreversibly to proteins in the kidney circulation to form advanced glycosylation end products that can form complexes that contribute to renal damage by stimulation of fibrotic growth factors [69].

HFD induces alteration of renal lipid metabolism by an imbalance between lipogenesis and lipolysis in the kidney, as well as systemic metabolic abnormalities and subsequent renal lipid accumulation leading to renal injury [70]. There is an association between CKD and insulin resistance that contributes to increased VLDL production and decreased HDL levels, both of which are considered risk factors for the development of kidney dysfunction and markers for progression of CKD [71].

In addition HFD resulted in hyperinsulinemia, activation of the renin-angiotensin system, glomerular hyperfiltration and structural changes in the kidney that may be the precursors of more severe glomerular injury associated with prolonged obesity [72].

The oral administration of L-carnitine (Table 4) shows that serum concentration of urea, uric acid and creatinine were highly significantly decreased. The effect of L-carnitine on renal lipid metabolism could serve as a new therapeutic approach, as it counters the renal changes associated with metabolic syndrome [73]. Hence, L-carnitine has beneficial effects on renal function.

Although there is still debate on the significance of uric acid as a risk factor for cardiovascular disease, many physicians do consider elevated uric acid to be a component of the metabolic syndrome. There is little support for an independent causal role for serum uric acid in the development of CHD. However, uric acid may provide useful prognostic information in subjects with hypertensive vascular disease, suggesting that the influence of uric acid on CHD is explained by the secondary association of it with other risk factors such as dyslipidemia, hyperinsulinaemia and obesity [74].

The oral administration of HMF extract (Table 4) clarified that serum concentration of urea; uric acid and creatinine were significantly decreased. Rhubarb results in lowering the serum creatinine level causing decrease in urinary protein excretion, attenuation of lipid derangements, decreased oxygen consumption and the hypertrophy of the remnant kidney [75]. Rhein, another ingredient in rhubarb of HMF, improves cell metabolism through glucose transporter-1: it decreases cell hypertrophy, indicating that there are multiple active ingredients even in a single herbal medicine involved in the multiple therapeutic effects of rhubarb in CKD, and suggesting that HMF might delay the progression of renal failure.

The herbal drugs containing tannins have a uremic-toxin-decreasing action, whereas rhubarb's tannins significantly improved BUN creatinine, glomerular filtration rate and renal blood flow [76]. In this respect rhubarb has proven effective as a diuretic in rabbit models, apparently by blocking sodium-potassium ATPase in the renal medulla [77].

The HMF therapy retains the balance between lipogenesis and lipolysis in the kidney to counteract the obesity-associated renal damage, in addition to maintaining cellular hydration due to laxative effect of senna, which is associated with diuretic effect of rhubarb, collectively leading to improvement in renal blood flow and consequent improvement in kidney function.

### Effect of treatments on glucose and insulin of HFD rats

Our results showed that a high fat diet results in significant increase in serum glucose, insulin level and insulin resistance (Table 5), which parallels the results obtained from **Zhang et al. **[78]. Diminished hepatic and muscular uptake of glucose produced hyperlipidemia due to increased fat mobilization from adipose tissue and resistance to the antilipolytic actions of insulin. Impaired insulin action is associated with an oversupply of lipids. This increased availability led to either elevated lipid stored in insulin target tissues (e.g. muscle, liver adipose) or increased plasma FFA or triglyceride [79].

The weight loss in this study due to oral administration of L-carnitine is associated with hypoglycemia, as it promotes insulin sensitivity, thus lowering insulin resistance in obese rats, possibly by regulating the cell energy metabolism or reducing free fatty acids as shown also by **González-Ortiz et al. **[53] and [55].

Obesity is associated with endothelial dysfunction through direct mechanisms, as insulin resistance in association with diabetes mellitus and dyslipidemia, indirectly, by the production of adipokines and pro-inflammatory cytokines, induces oxidative stress that affect the role of endothelium in modulating vascular function structure [80].

Our findings (Table 5) indicated a significant decrease of serum glucose, insulin and its resistance in obese rats treated with extracts of HMF. Some prenylflavonoids, such as glycycoumarin, glycyrin, dehydroglyasperin C and dehydroglyasperin D, in licorice ethanolic extract, are effective in preventing and ameliorating diabetes, abdominal obesity and preventing hypertension and metabolic syndrome [81].

In addition, hypoglycemia induced by T. chebula is probably mediated through enhanced secretion of insulin from the beta-cells of the pancreatic islets or through an extra pancreatic mechanism. Moreover, T. chebula may reduce the effect of inflammatory cytokine release during diabetes which may be one of the causative agents for the insulin resistance [69].

The hypoglycemic action of thymoquinone due to Nigella sativa in HMF could be partly due to preservation of β-cell integrity of the pancreatic islets causing a significant increase in insulin secretion. It also sensitized hepatocytes to the action of insulin. Furthermore, NS treatment exerts a therapeutic protective effect on diabetes by decreasing oxidative stress, preserving pancreatic beta-cell integrity [21].

### Effect of treatments on hepatic enzymes and lipid peroxidation in HFD-rats

The current data showed a significant increase in the activity of enzymes AST and ALT in the obese compared with normal rats (Table 4). Liver is bombarded by the free fatty acids (FFA) that pour out of the adipose tissue into the portal blood. This can directly cause inflammation within the liver cells, which then release further pro-inflammatory cytokines, leading to more hepatocyte injury and affecting the integrity of liver cells [82].

Administration of L-carnitine produces a significant lowering effect in the activity of AST and ALT in obese rats, a result in agreement with **Yapar et al. **[83]. Improvement of hepatocyte integrity does not seem to be limited to its obligatory role in the transmembrane import of fatty acids for mitochondrial β-oxidation, but L-carnitine prevents lipotrope methyl group wastage and increased production of polyamines with known immunomodulatory properties. Additionally L-carnitine can directly modify cytokine responses and reduce TNF-production. Moreover, lipid peroxidation is significantly blunted by oral administration of L-carnitine [84].

The present results demonstrate that the mixture of HMF showed a significant decrease in the activity of both AST and ALT (Table 4) agreeing with the resulted obtained by **Celik **and **Isik **[19].

D-limonene and β-myrcene in fennel *(Foeniculum vulgare*) has a potent hepatoprotective action; D-limonene increases the concentration of liver GSH which is required by several enzymes that participate in the formation of the correct disulfide bonds of many proteins. Polypeptide hormones participate in the metabolism of xenobiotics. β-myrcene elevates the levels of apoproteins CYP2B1 CYP2B2, which are subtypes of P450 enzyme system that catalyse the oxidative metabolism of a wide variety of exogenous chemicals including drugs, toxins, and endogenous compounds such as fatty acids [85]. Nigella sativa and licorice root extract of HMF possesses hepatoprotective effects in some models of liver toxicity [86].

HFD generates oxidative stress in obese rats as shown by a marked increase in the levels of MDA and a distinct diminution in hepatic GSH, as well as activities of the antioxidant enzyme catalase. All showed reduced activity in hyperlipidemic rats (Table 5).

Hyperglycemia in the HFD group activates different pathways leading to increased oxidative stress. Increased activity of the polylol pathway inhibition of the pentose phosphate pathway as a result of hyperglycemia resulted in decreased intracellular levels of NADPH, which is required for regeneration of GSH from its oxidized form GSSG [87]. The net result was non-enzymatic disruption of H_2_O_2 _and increased levels of cellular superoxides, hydroperoxides, hydroxyl radicals as well as other radicals.

In addition oxidative stress may be increased in metabolic syndrome due to dyslipidemia resulting from increased levels of FFA and TGs that led to increased formation of foam cells, rendering LDL less dense and more vulnerable to oxidation and uptake by macrophages [88].

Our results indicated that L-carnitine produced a significant inhibition of MDA production and a significant increase in GSH and activity of catalase (Table 5). L-carnitine reduces significantly the content of thiobarbituric acid reactive substances (TBARS), and causes marked increase in activity of catalase in skeletal muscles of obese rats [55]. Moreover L-carnitine favorably modulates oxidative stress causing a reduction in oxidized LDL cholesterol levels [89].

L-carnitine effectively protects and improves mitochondrial function in vivo: it acts as an antioxidant, so by inhibiting ROS it protects the vascular endothelial tissues against oxidative damage in hypertension [90]. Thus, L-carnitine treatment effectively protected the liver tissue against oxidative damage and showed marked improvement in its antioxidant status.

The current results demonstrated an antioxidative effect of HMF, as indicated by increased GSH level and hepatic catalase activity in comparison with obese rats (Table 5). Administration of aqueous extract of T. chebula effectively modulated oxidative stress and enhanced antioxidant status in the liver [91], which may be due to the total polyphenol content, expressed as gallic acid. T. chebula extract has the potential to play a role in the prevention of oxidative damage in living systems which can be attributed to its membrane stabilizing activities [92]. It has stronger antioxidant activity than α-tocopherol, due to the presence of hydroxybenzoic acid derivatives, flavonol aglycones and their glycosides.

Glabridin, a natural polyphenolic isoflavone antioxidant from licorice root extract in HMF possesses potent free radical scavenging activity that could promote a decrease in lipid peroxidation and could protect LDL from oxidation [93]. This would occur via a direct interaction with the lipoprotein and/or an indirect effect through accumulation in arterial macrophages. Therefore licorice represents a potent nutrient, which can attenuate the development of atherosclerosis, secondary to its antioxidant properties against lipids peroxidation in arterial cells [94].

Polyphenols, the main compound of N. sativa oil in HMF, have many biological properties. They possess powerful antioxidative components, which can inhibit membrane lipid peroxidation [28], and their administration exerts a therapeutic protective effect by decreasing oxidative stress.

*Foeniculum vulgare *extract, another constituent of the HMF, has potent antioxidant activity, as has been documented with various antioxidant assays. These activities include total antioxidant, free radical scavenging, superoxide anion radical scavenging, and hydrogen peroxide scavenging [95]. Antioxidant activity of the anthraquinones of rhubarb in the HMF may help protect against lipid peroxidation and free radical damage; its extracts will probably be useful for the development of safe food additives [12].

This study showed the antioxidant effect of dietary supplement represented by L-carnitine and free radical scavenger activities of herbal supplements represented by T. chebula, licorice, fennel, aniseed and Nigella sativa extract in the HMF.

Based on these broad observations, we suggest that high fat diet-induced obesity resulted in deleterious effects in kidney and liver tissues. HMF extract or carnitine supplementation counteracted the injuries, and ameliorated or normalized most the biochemical parameters.

Collectively obesity is associated with cardiometabolic complications, including insulin resistance, dyslipidemia, cardiovascular disease (CVD). When comparing therapies for obesity, both L-carnitine and HMF have shown improvements in body weight, lipid profile, glucose levels, liver, and kidney and cardiac marker of function, as well as insulin resistance and oxidative stress markers.

It can be concluded that in this study HFD induced a model of obesity associated with high body weight, hyperlipaemia, hyperinsulinemia, insulin resistance, hepatic oxidative stress, associated hepatic, cardiac renal function marker disturbance in rats. The obesity and its associated problems in this study could be ameliorated in different degrees by using L-carnitine or HMF extract.

The study also showed that herbal extract mixture significantly improved body weight, cholesterol, LDL triglyceride concentrations, creatinine, urea, ALT, oxidative stress, resulting from the high-fat diet. So it also had hypolipidemic, renoprotective, hapatoprotective, antioxidant and antiobesity effect.

It could be helpful to use HMF in conjunction with drugs; herbal extract might be a safer combination to surmount the overweight state. Positive results from such trials would confirm the medicinal usefulness of empirical combinations of traditional Egyptian medicines.

## Competing interests

The authors declare that they have no competing interests.

## Authors' contributions

KA and MN carried out experimental work; biochemical analysis, statistical analysis, interpretation and discussion of results related to their part of the work. KA, design and planning of the study; drafting and revision of the manuscript. All authors read and approved the final manuscript.
